# The Favorable Prognostic Factors for Superior Sulcus Tumor: A Systematic Review and Meta-Analysis

**DOI:** 10.3389/fonc.2020.561935

**Published:** 2020-10-20

**Authors:** Xiaohu Hao, Zihuai Wang, Diou Cheng, Jian Zhou, Nan Chen, Qiang Pu, Lunxu Liu

**Affiliations:** ^1^Department of Thoracic Surgery, West China Hospital, Sichuan University, Chengdu, China; ^2^West China School of Medicine, Sichuan University, Chengdu, China

**Keywords:** superior sulcus tumor, non-small cell lung cancer, prognostic factors, meta-analysis, systematic review

## Abstract

**Background:** Superior sulcus tumor is a rare non-small cell lung cancer with poor prognosis. Exploring the potential prognostic factors of patients with superior sulcus tumor and adopting individualized treatment for patients with different prognostic factors are of great significance for the prolongation of patients' lives. To figure out the prognostic factors of upper sulcus tumors, a meta-analysis was conducted.

**Method:** We searched all the articles published until January 2020 in PubMed, Embase, and Web of Science databases, and the search strategy included the following terms, combining superior sulcus tumor and prognosis. Hazard ratio (HR) with associated confidential interval (CI) was evaluated for the purpose of investigating prognostic factors for superior sulcus tumor. STATA 16.0 was used for analysis of extracted data and assessment of publication bias.

**Result:** Fifteen eligible studies, which had 1,009 patients with superior sulcus tumor, were included in this meta-analysis. The studies were published between 1994 and 2018, and the patient recruitment periods ranged from 1974 to 2016. The median follow-up time ranged from 18 to 95 months. The meta-analysis indicated that lower T stage (HR, 1.63; 95% CI, 1.35–1.97), lower N stage (HR, 3.08; 95% CI: 2.37–3.99), negative surgical margin (HR, 0.25; 95% CI, 0.17–0.38), and pathologic complete response (HR, 0.55; 95% CI, 0.39–0.77) were favorable prognostic factors.

**Conclusion:** We found that T stage, N stage, surgical margin, and pathologic complete response are prognostic factors for superior sulcus tumor. To reach a better long-term survival, patients with these negative prognostic factors may need a more aggressive treatment, while more studies should be conducted to further validate these results and explore a more effective treatment.

## Introduction

Superior sulcus tumor, involving the apex of the lung, is a rare subgroup of the non-small cell lung cancer (NSCLC). In 1932 (1), the radiologist Pancoast HK from Philadelphia first described this particular type of tumor accounting for <5% of NSCLC, which was the beginning of the report on Pancoast tumor.

Before the 1950s (2), the superior sulcus tumor was considered unresectable. In 1956, preoperative radiotherapy followed by surgery was first applied by Chardack and Maccallum (3), and the patient survived for more than 5 years. In 1961, preoperative radiotherapy followed by surgery (PRT-S) was demonstrated to have a higher 5-year survival rate (about 30%) (4). In the 1990s (5), two comparable prospective studies from the Southwest Cancer Research Group/North American study group (SWOG9416/Intergroup 0160) and the Japan Clinical Oncology Group (JCOG) (6) showed that preoperative chemoradiotherapy followed by surgery (PCRT-S) resulted in a 5-year survival rate of 44 and 56%, respectively, and it became the standard treatment recommended by the National Comprehensive Cancer Network (NCCN) guidelines.

In the past few decades, the 5-year survival rate of the superior sulcus tumor has increased a lot, but still many patients died due to recurrence and metastasis. The identification of prognostic factors is the key point to prolong the life of the patients. Although many studies have investigated the prognostic factors of superior sulcus tumors, the population was limited in each study, and the conclusions were inconsistent. This article was designed to explore and summarize the prognostic factors of the superior sulcus tumor by meta-analysis.

## Methods

### Search Strategy

This meta-analysis was conducted according to the Preferred Reporting Items for Systematic Reviews and Meta-Analyses (PRISMA) guidelines (7).

PubMed, Embase, and Web of Science databases were searched for all articles published till January 2020 by two authors (Xiaohu Hao and Zihuai Wang). The search strategy was comprised of [(superior sulcus) OR Pancoast] AND prognosis. No protocol has been previously conducted.

### Inclusion and Exclusion Criteria

Two authors (Xiaohu Hao and Zihuai Wang) conducted a study selection independently according to the PRISMA guidelines. Any discrepancies between the two investigators were resolved by discussing with a third reviewer (Diou Cheng). Eligible studies meeting the following criteria were included: (1) participants in the studies were diagnosed with superior sulcus tumor by pathology; (2) studies comparing survival outcome with different possible prognostic factors; (3) studies reporting the sufficient data of overall survival (OS) or disease-free survival (DFS) (4) on the randomized controlled trials (RCT) or observational studies published from inception to January 2020 in English. The exclusive criteria were as follows: (1) the survival outcomes were unavailable or could not be ascertained; (2) publication type other than original article; (3) participants in the studies were diagnosed with NSCLC other than superior sulcus tumor. No sample size restrictions were used. If there were repeated data (patients), the largest sample was selected.

### Quality Assessment

The Newcastle–Ottawa Scale (NOS) was used to evaluate the quality of original non-randomized studies. The scale includes three aspects of evaluation: selection methods of patients, comparability between groups, and assessment methods of outcome. The high-quality studies were defined as with at least eight stars; studies with at least five stars were included in our meta-analysis.

### Data Extraction

Data were independently extracted by two authors (Xiaohu Hao and Zihuai Wang) from all of the included studies. Any discrepancies between the two authors were resolved by discussing with the third reviewer (Diou Cheng). The following information were obtained in the original articles: (1) publication data including author, published year, and nationality of patients; (2) experimental data including study design, number of patients, inclusion period, follow-up time, chemotherapy regimen, radiotherapy regimen, and surgical approach; (3) demographic data including age, sex, and race; (4) clinicopathologic features including nodal status, pathologic complete response, completeness of resection, and T stage; and (5) survival data including OS, DFS, and hazard ratio (HR).

### Statistic Methods

STATA Version 16.0 was used for the analysis. In order to compare the prognosis of the patients, HR with 95% CI of 5-year OS of patients with or without certain possible prognostic factors was directly extracted from the text or calculated from the Kaplan–Meier survival curve. Heterogeneity of different studies was examined using the Cochrane Q test by calculating the *I*^2^ value. If the significant heterogeneity (*I*^2^ > 50% or *P* < 0.05) was observed, then the random effect model would be used to calculate the HR; otherwise, the fixed effect model would be used. Sensitivity analysis was performed by sequentially removing each study, and publication bias was tested by the Begg's test.

## Results

### Eligible Studies and Characteristics of Studies

**Figure 1:**
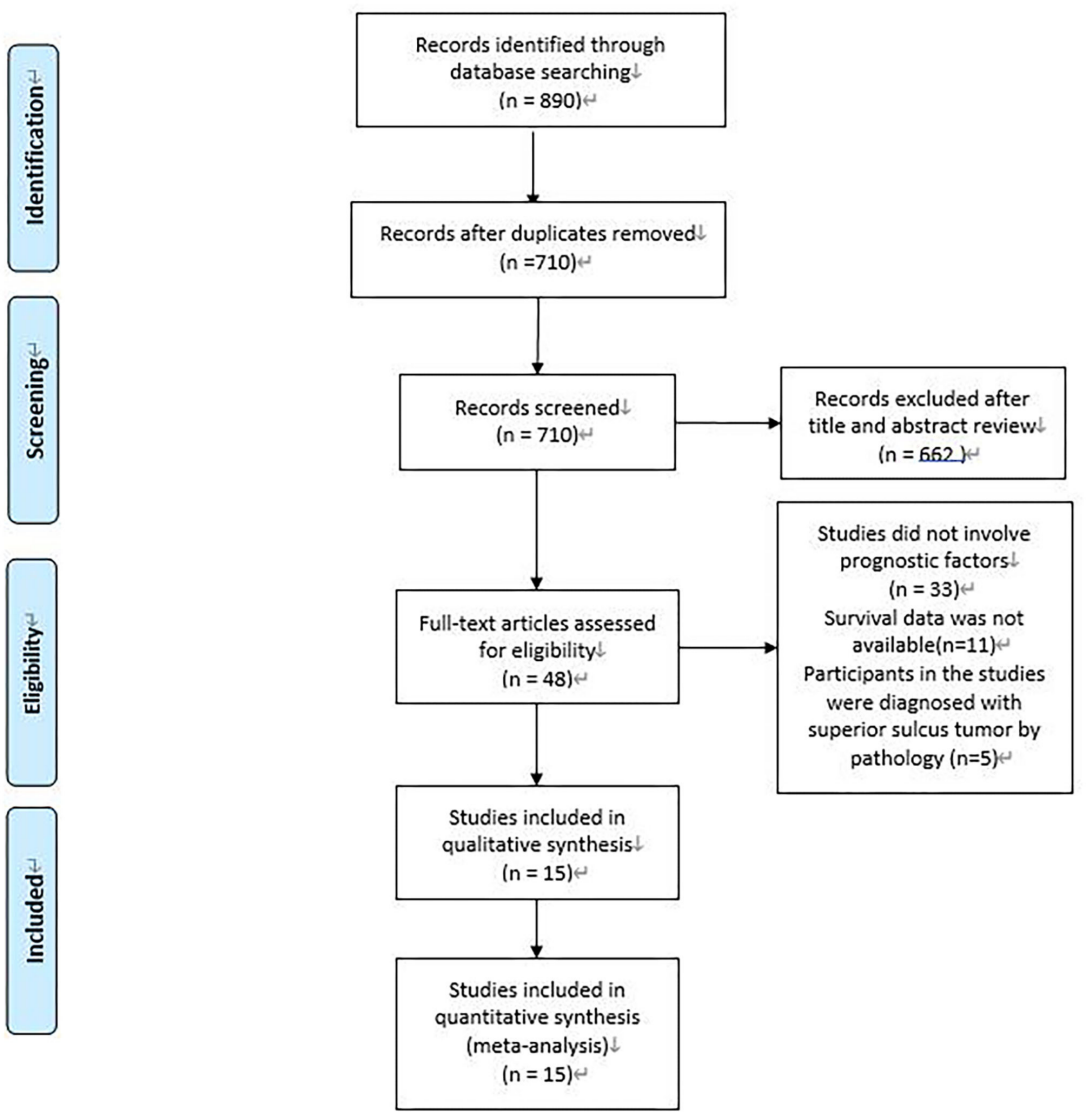
Flow chart of the identification of relevant studies.

**Figure 2:**
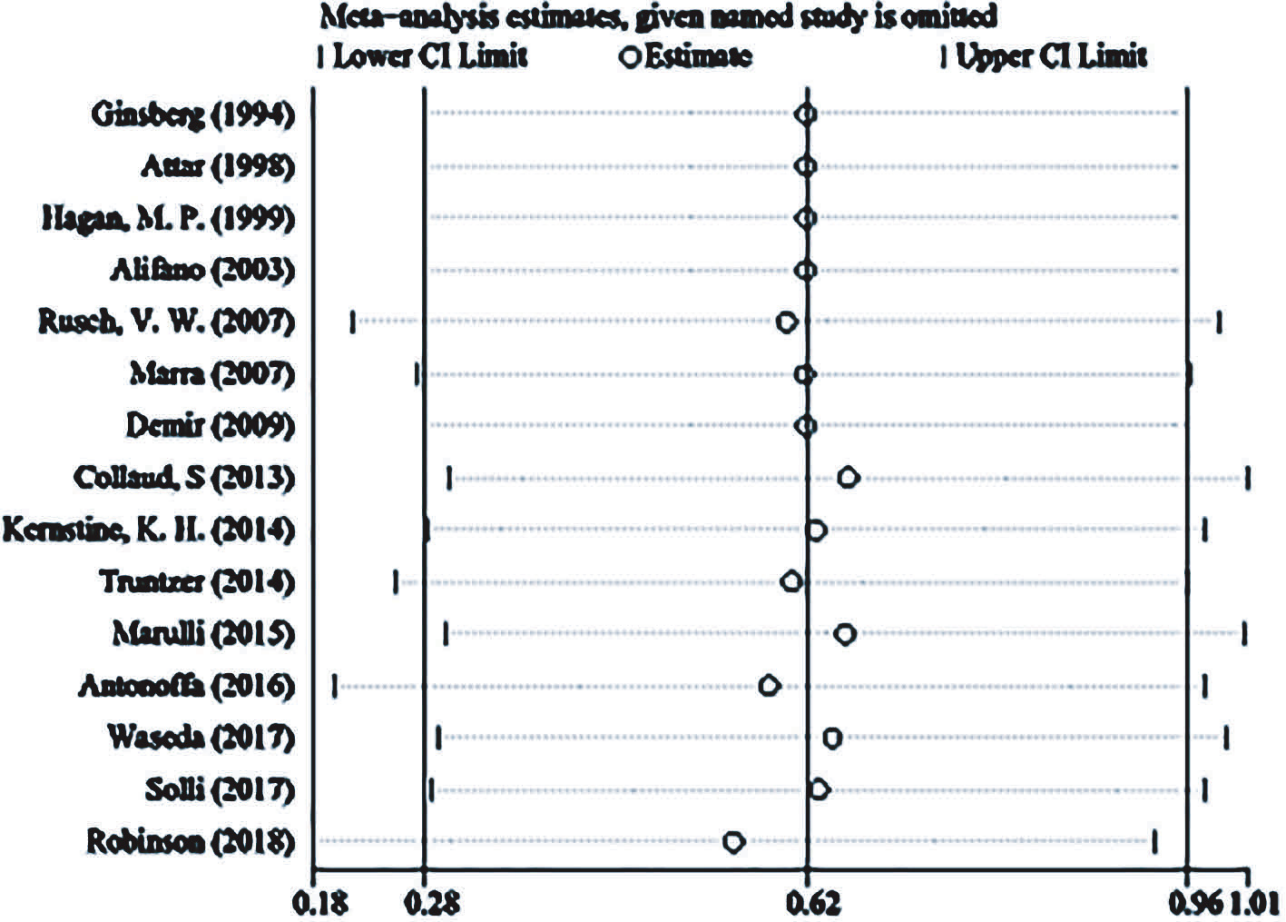
Sensitivity analysis in studies assessing prognostic factors of superior sulcus tumor.

**Figure 3:**
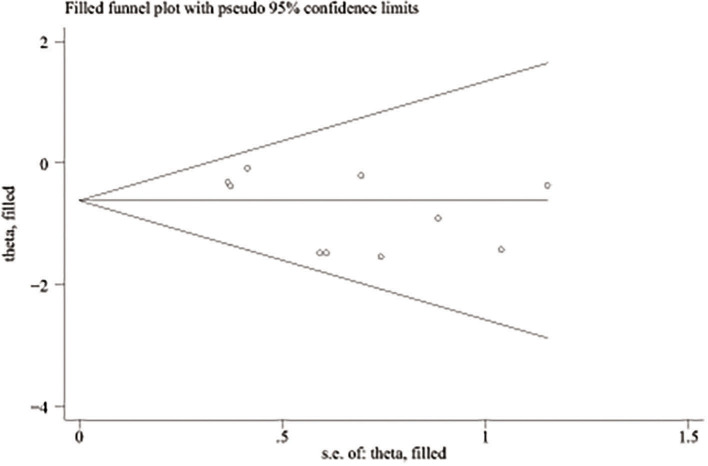
Publication bias of relevant studies.

A total of 890 potentially relevant studies were included in this study according to the initial search strategies. During the systematic review process, 180 studies were excluded because of repetition, 662 studies were excluded after reviewing the title and the abstract, and 33 studies were excluded owing to the unavailable survival data and improper participants. Eventually, 15 eligible studies, which had 1,111 patients were included in the meta-analysis (Figure 1). Seven studies (5, 8–13) were evaluated as high quality, and the other studies (13–22) had at least six stars (Table 1). The studies were published between 1994 and 2018, and the patient recruitment periods ranged from 1974 to 2016. The median follow-up time was between 18 and 95 months. Most studies were retrospective, except for two prospective studies (5, 23). Because of the large time span, the chemotherapy and radiotherapy schemes in different studies were quite different, and survival rates varied widely in different studies, with 5-year OS ranging from 18 to 64%. All surgeries were performed by thoracotomy and lobectomy. Details about the eligible studies are reported in Table 2. Sensitivity analysis was conducted by sequentially removing each study, and the results indicated a consistent result among all studies (Figure 2). Publication bias was assessed with the Begg's test, and no publication bias was found (*p* = 0.616) (Figure 3).

**Table 1:** Result of the quality assessment by the Newcastle–Ottawa Scale (NOS).

**Study**	**Selection**				**Comparability**	**Outcome**			**Total**
	**Exposed**	**Non-exposed**	**Ascertainment of**	**Outcome of**		**Assessment of**	**Length of**	**Adequacy of**	
	**cohort**	**cohort**	**exposure**	**interest**		**outcome**	**follow-up**	**follow up**	
Alifano	*	*	*	*	*	*	*	*	8
Antonoff	*	*	*	*	*	*	*	–	7
Attar	*	*	*	*	*	*	*	–	7
Collaud	*	*	*	*	*	*	*	*	8
Demir	*	*	*	*	*	*	*	–	7
Ginsberg	*	*	*	*	–	*	*	–	6
Hegan	*	*	*	*	*	*	*	*	8
Kernstine	*	*	*	*	*	*	*	*	8
Marra	*	*	*	*	*	*	*	*	8
Marulli	*	*	*	*	*	*	*	*	8
Robinson	*	*	*	*	*	*	*	–	7
Rusch	*	*	*	*	*	*	*	*	8
Solli	*	*	*	*	*	*	*	–	7
Trunzter	*	*	*	*	*	*	*	–	7
Waseda	*	*	*	*	*	*	*	–	7

**Table 2:** Characteristics of the included studies.

**First author**	**Year**	**Country**	**Sample size**	**Recruitment period**	**Median follow-up (month)**	**Study design**	**Quality assessment**
Attar	1998	USA	105	1995–1997	NA	Retrospective	7
Alifano	2003	France	67	1988–2002	50 (3–176)	Retrospective	8
Antonoff	2016	USA	102	1988–2013	18	Retrospective	7
Collaud	2013	Canada	48	1991–2012	26 (0–151)	Retrospective	8
Demir	2009	Turkey	65	1994–2007	28 ± 34 (2–148)	Retrospective	7
Ginsberg	1994	USA	124	1974–1991	NA	Retrospective	6
Hegan	1999	USA	73	1975–1992	NA	Retrospective	8
Kernstine	2014	USA	46	2003–2007	62	Retrospective	8
Marra	2007	Germany	31	1993–2004	40 (24–134)	Prospective	8
Marulli	2015	Italy	56	1994–2013	95 (4–187)	Retrospective	8
Rusch	2007	USA	110	1995–1999	NA	Prospective	8
Robinson	2018	USA	102	1994–2016	72.5	Retrospective	7
Solli	2017	Italy	94	1998–2013	23 (0–176)	Retrospective	7
Trunzter	2014	France	42	2000–2010	44.1 (0–128)	Retrospective	7
Waseda	2017	Austria	46	1998–2013	42.3 (alive patients) 49 (patients still alive without any recurrence)	Retrospective	7

### Tumor Stage

**Figure 4:**
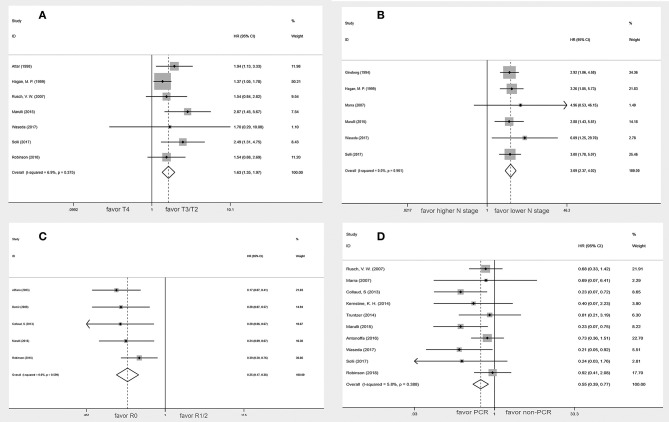
Forest plot of the potential prognostic factors of the superior sulcus tumor. **(A)** Tumor stage; **(B)** lymph nodes status; **(C)** Status of surgical margins; **(D)** Pathologic complete response.

Seven studies (5, 10, 12, 15, 20–22) were included in the analysis of the relationship between T stage and survival. In these studies, patients were divided into two groups according to the T stage (T4 vs. T3). Only one study (21) included a small number of T2 patients (T4 vs. T3/2). Among the 577 patients, 203 patients (35.2%) were T4, 367 patients (63.6%) were T3, and 7 patients (1.2%) were T2. The heterogeneity was assessed by *I*^2^ statistic and was 6.9% (*P* = 0.375). Thus, the fixed-effect model was validated. A meta-analysis showed a statistically significant difference between T3 and T4 patients (HR, 1.63; 95% CI, 1.35–1.97) (Figure 4A). The results indicated that T4 was associated with a worse OS.

### Lymph Node Status

In six studies (10, 12, 17, 21–23), lymph node status was classified as N2-3 vs. N0-1 in two studies (17, 23), N+ vs. N0 in three studies (10, 21, 22), and N2-3 vs. N1 in one study (12). These six studies, which included 420 patients, indicated that statistically significant difference in OS was found in the higher N stage groups compared with the lower N stage groups (HR, 3.09; 95% CI: 2.37–4.02) without heterogeneity (*I*^2^ = 0.0%, *P* = 0.961) (Figure 4B).

### Status of Surgical Margins

The status of surgical margins as a prognostic factor for superior sulcus tumor was reported in five studies (8, 9, 12, 16, 20). Among the 328 patients, 275 (83.8%) had negative surgical margins, and 53 (16.2%) had positive surgical margins. The present meta-analysis indicated that the negative surgical margin patients had a better overall survival rate than the positive surgical margin patients (HR, 0.25; 95% CI, 0.17–0.38) without a significant heterogeneity (*I*^2^ = 0.0%, P = 0.599) (Figure 4C).

### Pathologic Complete Response

Data on pathologic complete response were available from 10 studies (5, 9, 11, 12, 14, 19–23). Among the 466 patients, 232 (50.0%) achieved a pathologic complete response, and 234 (50.0%) did not achieve a pathologic complete response. Pathologic complete response was associated with better overall survival (HR, 0.55; 95% CI, 0.39–0.77). This analysis revealed no heterogeneity (*I*^2^ = 5.8%; *p* = 0.388) (Figure 4D). The subgroup analysis of five studies (9, 14, 19, 21, 22) using the seventh edition of TNM staging published after 2013 suggested that pathologic complete response was also associated with better overall survival (HR, 0.48; 95% CI, 0.29–0.79).

## Discussion

In the last century, treatment strategies had evolved in the management of superior sulcus tumor, and the 5-year survival rate increased a lot. Nevertheless, a large number of patients died due to metastasis and recurrence. Due to the limited patient number, the available data for long-term survival of SST were rare. However, a few articles discussed the prognostic factors for SST patients, which could distinguish patients into a distinct subgroup, and developed a more aggressive treatment plan.

According to our results, four prognostic factors were discovered, including T stage, surgical margin status, lymph node status, and pathologic response. SST patients are usually diagnosed in the T3 or T4 stages, about 75% patients in the T3 stage, and 25% in the T4 stage (11). A surgery-based multimodality treatment is recommended in the T3 and marginally resectable in the T4 patients. T4 patients' tumors have larger size and are likely to invade the adjacent tissue. A higher rate of positive surgical margin is also observed in T4 patients and may lead to inferior survival in SST patients.

Lymph node status is another important prognostic factor. Among stage IIIa N2 NSCLC patients, postoperative radiotherapy is recommended in the positive mediastinal lymph node field. However, a few included articles validated postoperative radiotherapy either in positive surgical margin or N2 metastatic SST patients. Additional aggressive treatment methods might be performed on these patients. Pathologic complete response (pCR) can be achieved in about 20% of the patients after preoperative chemoradiotherapy, which was thought to be a prognostic factor. However, seven of the included articles found no significant prognostic differences in pCR patients. Two reasons may lead to this result: first is the detection of pCR, which was defined as no tumor cell left in the previous foci and only a lymphocyte can be found. The second reason is the different treatment strategy, as some centers tended to take surgery first followed by postoperative chemo/chemoradiotherapy.

Though the treatment strategy recommended in the NCCN guideline was preoperative chemoradiotherapy plus surgery and postoperative chemotherapy, the real-world statistics varied a lot among different centers. Solli et al. (21) validated preoperative treatment in only half of the patients (51%) with neoadjuvant chemotherapy being the fundamental treatment. Considering the high risk of complication due to chemo and radiotherapy, they took surgery as the first step and showed the comparable survival outcomes as well as the complete resection rate (90%).

From the last century, although the treatment method has changed a lot, en bloc resection has always been the key method in the treatment of superior sulcus tumor. In the previous studies, the status of surgical margins was a significant prognostic factor (12, 17, 24). Our meta-analysis also suggested that positive surgical margins were a poor prognostic factor. The tumor cells in positive surgical margins become the potential sources for local recurrence. Because of the violation of the adjacent tissues or organs, completeness of resection is difficult for superior sulcus tumor, but it was reported that complete resection rate was over 80% due to preoperative chemoradiotherapy (12). T stage and response to the preoperative neoadjuvant therapy are important influence factors to the complete resection.

We also tried to conduct a meta-analysis on the treatment methods of superior sulcus tumor, but due to the limited number of studies and different combinations of treatment methods, the results of the meta-analysis could not be obtained. Compared with PRT-S, PCRT-S had a significant improvement in local recurrence rate and 5-year survival rate. However, the distant recurrence became the major reason for about 80% recurrence (11). SWOG 9416 tried to add two cycles of consolidate chemotherapy based on etoposide and cisplatin after surgery. However, patients were unable to complete the prescribed adjuvant chemotherapy. SWOG S0220 added two cycles of consolidate chemotherapy based on docetaxel. The 3-year overall survival was 61%, and progression-free survival was 56%. Although more R0 and local control rates have been achieved, distant metastases, especially brain metastases, still seriously threaten the survival of patients. More effective systemic treatments and management of brain metastasis need to be explored. The efficacy of preoperative chemoradiotherapy followed by surgery and consolidation chemotherapy also needs to be confirmed by large clinical studies involving more patients.

There are some limitations in this article. Due to the small number of patients with superior sulcus tumors, most studies have taken longer to accumulate enough patients. As there were few related studies published, the time span of the included studies was also very large. There might be differences in the diagnosis and treatment of patients. Therefore, we intend to conduct a subgroup analysis of all prognostic factors according to different periods. However, due to the small number of studies, most of the studies had a large time span, and the staging version was not clearly indicated. Thus, subgroup analysis could not be performed. For the pathologic complete response with the largest number of studies, we conducted a subgroup analysis according to different periods, and the results showed that there was no difference between the two groups. In terms of tumor staging, although different staging versions were used in different periods, superior sulcus lung tumors were mainly classified as T3/T4 due to the invasion of surrounding tissues. There are a few differences between different versions, and there are a few changes in N staging. The definition of surgical margin has basically not changed. It is hoped that there will be more large-scale studies to confirm the results of this article in the future.

## Conclusion

By the meta-analysis, we found that lower T-stage, N-stage, negative surgical margin, and pathologic complete response were favorable prognostic factors for SST patients. The patients with negative factors may need more powerful treatment. More studies should be conducted to further validate these results and explore the more effective treatment.

## Data Availability Statement

All datasets presented in this study are included in the article/Supplementary Material.

## Author Contributions

XH, ZW, and LL conceptualized and designed the study. LL provided administrative support. XH and ZW were responsible for document retrieval. XH, ZW, and DC were in charge of the collection and assembly of data. JZ, NC, and QP analyzed and interpreted the data. All authors wrote and approved the final version of the manuscript.

## Conflict of Interest

The authors declare that the research was conducted in the absence of any commercial or financial relationships that could be construed as a potential conflict of interest.

## Supplementary Material

Supplementary Figure S1 Forest plot of the subgroup analysis for the pathologic complete response of the studies using the seventh edition of TNM staging published after 2013.
