# Supplementary figures and images for: The Favorable Prognostic Factors for Superior Sulcus Tumor: A Systematic Review and Meta-Analysis
Source: Front Oncol. 2020 Oct 20;10:561935. doi: 10.3389/fonc.2020.561935 (PMC7606951; doi:10.3389/fonc.2020.561935)

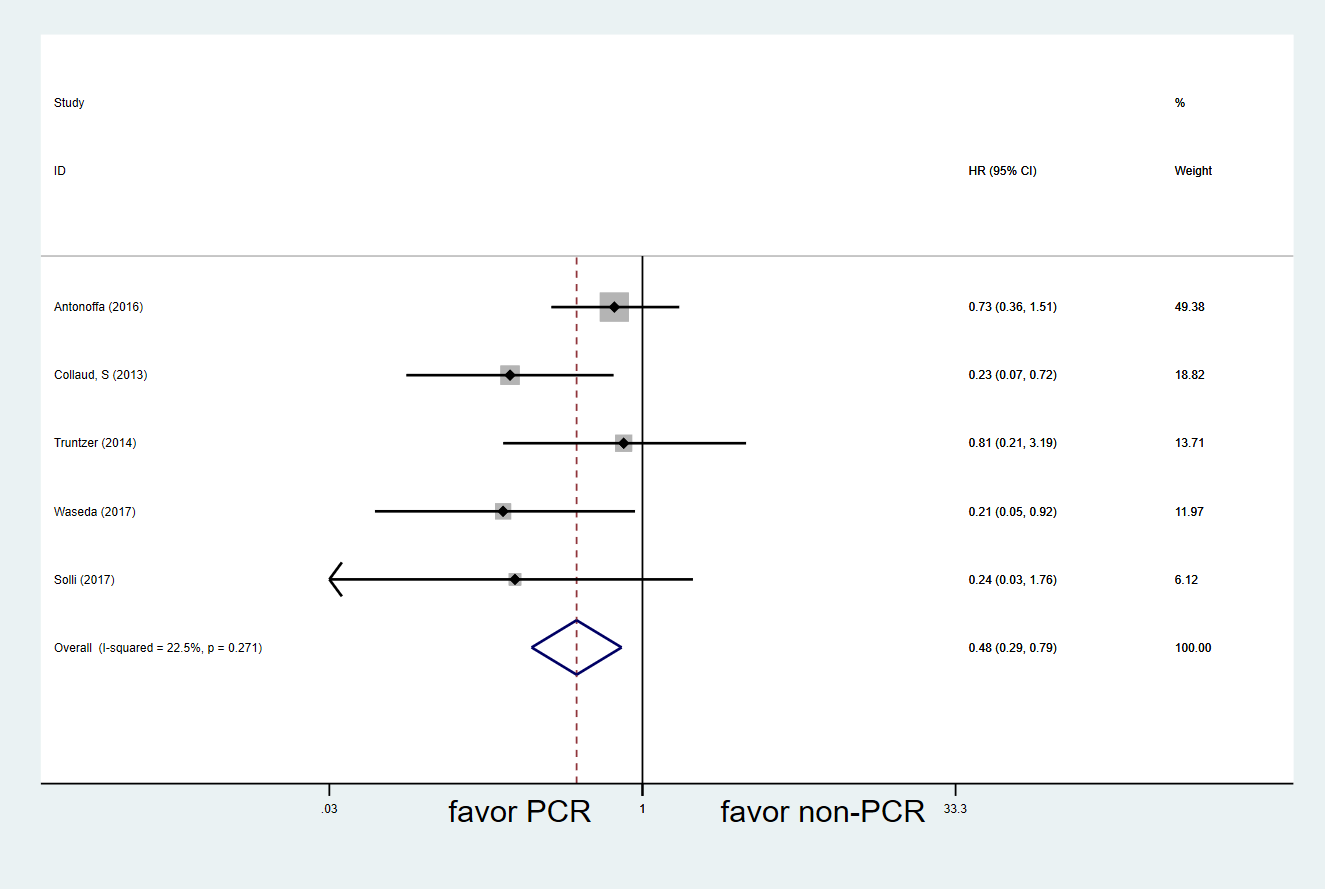

Supplement: Supplementary Figure S1 — Forest plot of the subgroup analysis for the pathologic complete response of the studies using the seventh edition of TNM staging published after 2013. [file Image_1.TIF]
